# The association between fibrinogen-to-albumin ratio (FAR) and adverse prognosis in patients with acute decompensated heart failure at different glucose metabolic states

**DOI:** 10.1186/s12933-022-01662-x

**Published:** 2022-11-12

**Authors:** Rong Huang, Qing Dai, Lei Chang, Ziyan Wang, Jianzhou Chen, Rong Gu, Hongyan Zheng, Lei Hu, Biao Xu, Lian Wang

**Affiliations:** 1 Department of Cardiology, Nanjing Drum Tower Hospital, The Affiliated Hospital of Nanjing University Medical School, Nanjing, 210008 Jiangsu China; 2 Department of Cardiology, Nanjing Drum Tower Hospital Clinical College of Nanjing Medical University, Nanjing, 210008 Jiangsu China; 3 Department of Cardiology, Nanjing Drum Tower Hospital Clinical College of Jiangsu University, Nanjing, 210008 Jiangsu China

**Keywords:** Fibrinogen-to-albumin ratio, Acute-decompensated heart failure, Major adverse cardiac and cerebral events

## Abstract

**Background:**

Circulating fibrinogen-to-albumin ratio (FAR) has been proposed as a novel inflammatory biomarker and a cardiovascular disease risk predictor. However, its prognostic value in patients with acute decompensated heart failure (ADHF) and different glycemic metabolic states remains ambiguous.

**Methods:**

A total of 1031 hospitalized patients with ADHF from January 2018 to May 2021 were included in the study. The primary endpoints were the major adverse cardiac and cerebral events (MACCEs). Patients were categorized into high-level FAR (FAR-H) and low-level FAR (FAR-L) groups based on the optimal cut-off value of FAR obtained from restricted cubic spline function analysis. The Kaplan–Meier plots and three multivariate-adjusted Cox proportional hazard models were used to determine the association between FAR and the risk of developing MACCEs in patients with ADHF at different glycemic metabolic states.

**Results:**

MACCEs occurred in 483 (46.8%) patients during a median follow-up time of 520 days. The optimal FAR cut-off value was 0.079. Upon analyzing the Kaplan–Meier plots, the incidence of MACCEs was significantly different between the FAR groups in all patients and patients with diabetes mellitus (p < 0.05). After adjusting for the confounding factors, the hazard ratio (HR) for MACCEs in the FAR-H group was 1.29 compared with the FAR-L group in all patients (Model 3: 95% CI 1.07–1.56, p = 0.007). Additionally, high FAR was associated with MACCEs in three multivariate Cox models (Model 1, HR = 1.52, 95% CI 1.17–1.96, p = 0.002; Model 2, HR = 1.46, 95% CI 1.13–1.89, p = 0.004; Model 3, HR = 1.48, 95% CI 1.14–1.92, p = 0.003) in DM patients. But no significant differences were found between the FAR groups for prediabetes mellitus (Pre-DM) and normal glucose regulation (NGR) using the three Cox models (all p-values were > 0.05).

**Conclusions:**

Elevated FAR was independently associated with poor prognosis in patients with ADHF and DM and thus could be used as a risk stratification tool and a potential therapeutic target in the future.

## Introduction

Although systemic inflammation has been considered a common pathophysiological characteristic of acute and chronic heart failure (HF) [1, 2], the effect of inflammation in HF has long been controversial. Several clinical trials on anti-inflammation and HF yielded controversial outcomes [3–6]. However, the outcome of the CANTOS trial [7] published in 2019 revealed chronic inflammation as a critical process in the pathogenesis of HF, leading to the exploration of more biomarkers screening patients with HF that may benefit from anti-inflammatory strategies. Various biomarkers were elevated in response to the activation of systemic inflammation in patients with HF [8], and it is more evident in acute HF [2].

Fibrinogen is a glycoprotein complex synthesized in the liver that takes part in the processes of inflammation and thrombosis [9, 10]. Previous studies found associations of fibrinogen with the risk of mortality in patients with coronary artery disease (CAD) [11]. Albumin is a protein synthesized in the liver and influences nutrient absorption, colloidal pressure, and systemic inflammation [12, 13]. Fibrinogen-to-albumin ratio (FAR) is the proportion of these two indexes, which is associated with poor prognosis in a variety of cancers [14–17]. The FAR was also directly associated with the severity of coronary artery calcification and adverse prognosis of the acute coronary syndrome, especially in patients with diabetes mellitus (DM) [18–20]. Furthermore, FAR was more sensitive and specific in predicting major adverse cardiovascular events (MACE) than fibrinogen and albumin alone [21, 22].

As common comorbidity of heart failure, DM creates a chronic low-grade inflammatory environment, leading to further impairment of cardiac structure and function [4, 23]. Patients with HF and DM had a worse prognosis than patients with HF alone [24, 25]. Nevertheless, reports are lacking on the relationship of FAR with the prognosis of acute-decompensated heart failure (ADHF). Therefore, this retrospective study aimed to evaluate the relationship between FAR and major adverse cardiac and cerebrovascular events (MACCEs) in patients with ADHF under different glucose metabolism states.

## Materials and methods

### Study population

**Fig. 1:**
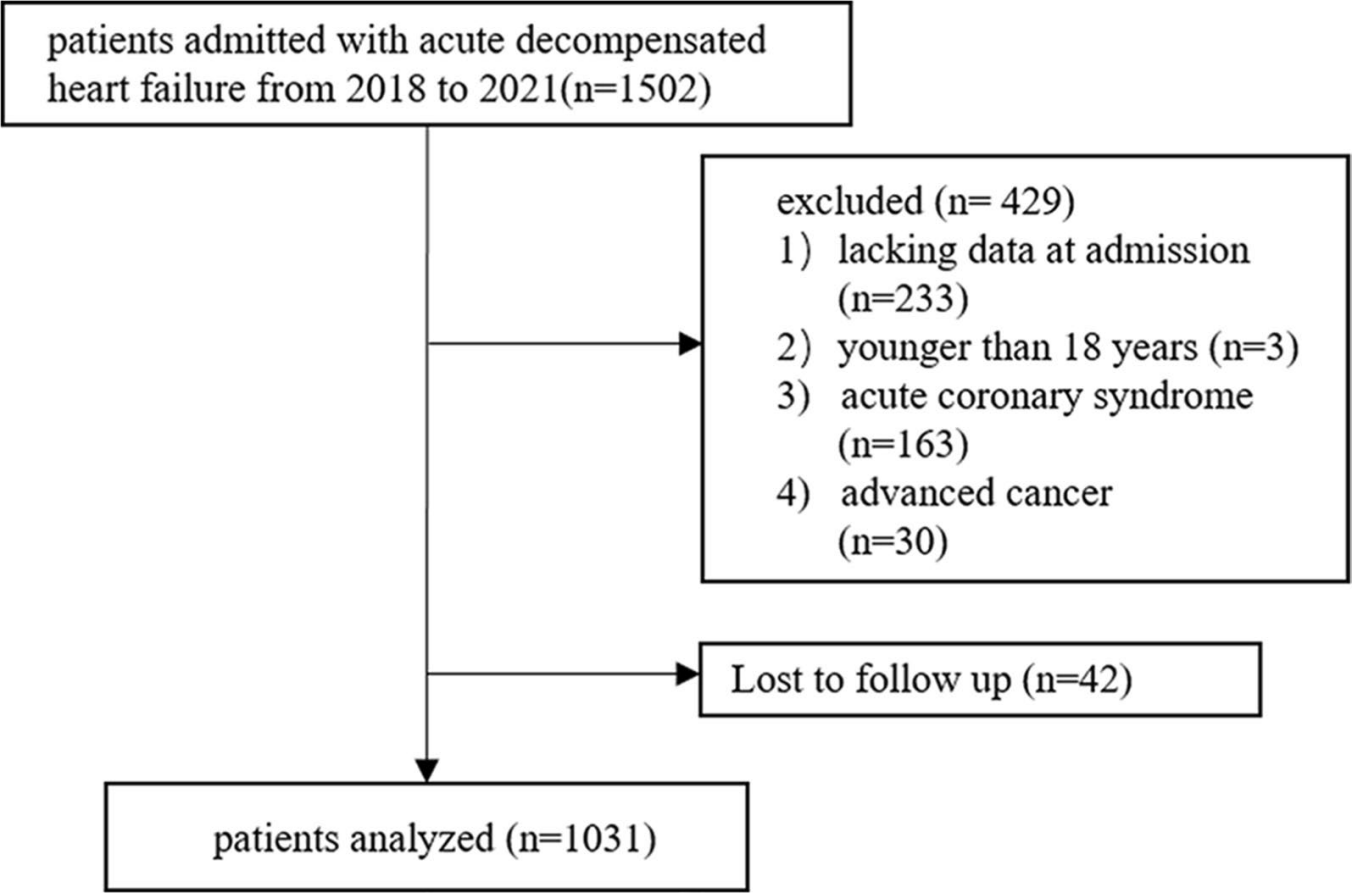
Flow diagram of patient selection

The single-center retrospective analysis of 1502 consecutive patients with ADHF admitted to Nanjing Drum Tower Hospital from January 2018 to May 2021 was carried out. The ADHF was defined using the 2021 ESC Guidelines for the diagnosis and treatment of acute and chronic heart failure [26]. The exclusion criteria were: (1) patients with missing baseline data, (2) patients who were younger than 18 years, (3) patients with acute coronary syndrome, (4) patients with advanced cancer, and (5) patients who were lost to follow-up. The inclusion criteria are detailed in Fig. 1. This study was performed following the Declaration of Helsinki and with the approval of the Ethics Committee of the Nanjing Drum Tower Hospital.

### Data collection and definitions

Trained physicians retrieved patient demographics, clinical history, anthropometric data, laboratory test results, echocardiographic data, and information on medication during hospitalization from the electronic medical records.

FAR was defined as the proportion of the plasma fibrinogen (g/L) to the plasma albumin level (g/L). Body mass index (BMI) was denoted as weight (kg)/(height [m])^2^. Based on the recommendations from the American Diabetes Association [27], DM was defined as fasting plasma glucose (FPG) of ≥ 7.0 mmol/L or glycated hemoglobin (HbA1c) of ≥ 6.5%, or the current use of hypoglycemic medications. Prediabetes mellitus (Pre-DM) was defined as 5.6 mmol/L ≤ FPG < 7.0 mmol/L or 5.7% ≤ HbA1c < 6.5%, and normal glucose regulation (NGR) was considered patients without pre-DM or DM. Hypertension was considered when there was a systolic blood pressure (SBP) of ≥ 140 mmHg and/or diastolic blood pressure (DBP) of ≥ 90 mmHg, any use of antihypertensive drugs, or having a history of hypertension. Hyperlipidemia was defined when the fasting total cholesterol (TC) was ≥ 240 mg/dL, low-density lipoprotein cholesterol (LDL-C) was > 160 mg/dL, TGs were ≥ 150 mg/dL, high-density lipoprotein cholesterol (HDL-C) was < 40 mg/dL, or lipid-lowering drugs were previously used.

The peripheral venous blood samples were initially collected after overnight fasting (> 8 h) and the different variables were determined in the laboratory. Fibrinogen, albumin (ALB), C-reactive protein (CRP), B-type natriuretic peptide (BNP), hemoglobin, hematocrit, uric acid (UA), creatinine, white blood cell count (WBC), FPG, HbA1c, total cholesterol, triglycerides, LDL-C, HDL-C, serum sodium, serum potassium, alanine aminotransferase (ALT), and aspartate transaminase (AST) were measured.

### Endpoints and follow-up

The primary endpoint was MACCEs, such as all-cause death, cardiovascular death, non-fatal myocardial infarction, non-fatal ischemic stroke, and chronic HF worsening.

Patients were followed up by telephone and/or during clinic visits every six months.

### Statistical analysis

Continuous variables were expressed as mean ± standard deviation (SD) or median [interquartile range (IQR)]. Categorical variables were expressed as numbers (percentages).

A restricted cubic spline regression analysis was performed to determine the optimal cut-off value of the FAR. Patients were categorized into two groups based on their FAR values [FAR-L (< 0.079) and FAR-H (≥ 0.079)]. Continuous variables were compared using analysis of variance (ANOVA) or the Kruskal–Wallis test. Categorical variables were compared using the χ2 test.

The cumulative event-free survival rate was calculated using the Kaplan–Meier plots and the log-rank test. Multivariate Cox proportional hazards models were applied to determine the associations of FAR with the incidence rates of MACCEs. Schoenfeld residuals were visually interpreted to validate the assumptions of proportional hazards before these analyses. The baseline variables with a p-value of < 0.1 or clinically significant were included in the Cox proportional hazards models. Multicollinearity was tested in the multivariate models with a threshold of variance inflation factor of < 5. Finally, three multivariate regression models remained: Model 1 with the adjustment for age, sex, BMI, SBP, DBP, and heart rate; Model 2 with the adjustment for variables included in Model 1 plus BNP, creatinine, WBC, sodium, UA, ALT, and left ventricular ejection fraction (LVEF); Model 3 with the adjustment for variables included in Model 2 plus history of hypertension, valvular heart disease, atrial fibrillation, and use of aldosterone antagonist, β-Blockers, ACEI/ARB/ARNI, or SGLT2i. Moreover, the prognostic value of FAR for MACCEs at different glucose metabolic states was also assessed by the models. Hazard ratios (HRs) were calculated and the results were reported as HRs and 95% confidence intervals (CIs). The low FAR was used as a reference in the three models. The correlation between FAR and cardiometabolic-related factors was analyzed using Spearman’s rank correlation. The subgroup analyses including age, sex, BMI, and glucose metabolism state were shown as the forest plot. All data were analyzed using R version 4.1.0, GraphPad Prism version 8.0.1, and SPSS for windows version 26. A P-value of < 0.05 was considered statistically significant.

## Results

### General characteristics of patients

The clinical characteristics of the participants relevant to the occurrence of MACCEs are listed in Table 1. The median follow-up time was 520 days. The proportions of patients who previously experienced non-ischemic cardiomyopathy (NIMD), pre-DM, NGR, hyperlipidemia, intake of angiotensin-converting enzyme inhibitor/angiotensin receptor inhibitor/ angiotensin receptor neprilysin inhibitor (ACEI/ARB/ARNI) were significantly lower in the MACCEs group. Further, DBP, ALB, hemoglobin, hematocrit, sodium, total cholesterol, FPG, and HDL-C were lower in the MACCEs group (all p-values were < 0.05). Additionally, the patients in the MACCEs group were older and had higher levels of FAR, HbA1C, CRP, BNP, UA, Creatinine, AST, the proportion of DM, and digoxin intake (all p-values were < 0.05).

**Table 1:** Baseline clinical characteristics and MACCEs

Characteristics	Total (n = 1031)	Non-MACCEs (n = 548)	MACCEs (n = 483)	p Value
Female, n (%)	393 (39.1)	202 (36.9)	191 (39.5)	0.376
Age (years)	71 (61, 79)	69 (58, 77)	73 (64, 81)	< 0.001
BMI (kg/m^2^)	24.1 (21.5, 27.1)	24.2 (21.5, 27.1)	23.9 (21.6, 27.1)	0.894
Heart Rate (bpm)	84 (69, 97)	80 (69, 96)	81(70, 98)	0.172
SBP (mmHg)	128 (114, 144)	128 (115, 144)	128 (113, 145)	0.727
DBP (mmHg)	75 (66, 86)	76 (67, 88)	74 (64, 85)	0.003
Medical history, n (%)				
Ischemic cardiomyopathy	390 (37.8)	199 (36.3)	191 (39.5)	0.286
None-ischemic cardiomyopathy	209 (20.3)	126 (23.0)	83 (17.2)	0.021
Valvular heart disease	203 (19.7)	102 (18.6)	101 (20.9)	0.355
Atrial fibrillation	446 (43.3)	225 (41.1)	221 (45.8)	0.129
Hypertension	625 (60.6)	323 (58.9)	302 (62.5)	0.240
Hyperlipidemia	88 (8.5)	59 (10.8)	29 (6.0)	0.006
Glucose metabolism state, n (%)				< 0.001
DM	505 (49.0)	230 (42.0)	275 (56.9)	-
Pre-DM	309 (30.0)	190 (34.7)	119 (24.6)	-
NGR	217 (21.0)	128 (23.4)	89 (18.4)	-
FAR	0.078 (0.064, 0.097)	0.076 (0.063, 0.092)	0.081 (0.065, 0.103)	0.002
Fibrinogen (g/L)	3.0 (2.5, 3.6)	3.0 (2.5, 3.5)	3.0 (2.4, 3.7)	0.283
ALB (g/L)	38.1 (35.4, 40.5)	38.8 (36.5, 41.0)	37.2 (34.4, 39.9)	< 0.001
FPG (mmol/L)	5.08 (4.58, 6.22)	5.02 (4.59, 6.05)	5.21 (4.61, 6.64)	0.058
HbA1c (%)	6.1 (5.6, 7.0)	6.0 (5.6, 6.8)	6.2 (5.7, 7.1)	< 0.001
Hemoglobin (g/L)	128 (111, 142)	132 (116, 145)	124.0 (105.0, 137.0)	< 0.001
BNP (pg/mL)	496 (212, 1120)	429 (192, 887.5)	649 (269, 1330)	< 0.001
CRP (mg/L)	4.6 (3.0, 12.0)	4.0 (2.8, 8.3)	5.6 (3.25, 19.6)	< 0.001
Hematocrit (%)	38.5 (33.8, 42.2)	39.4 (5.0, 42.9)	37.5 (32.2, 41.0)	< 0.001
UA (μmol/L)	430 (339, 541)	422 (329, 517)	441 (357, 577)	0.004
Creatinine (μmol/L)	84.7 (66.0, 112.0)	80.0 (64.0, 102.5)	88.3 (70.2, 127.4)	< 0.001
Sodium (mmol/L)	138.5 (135.5, 141.5)	138.9 (135.8, 141.6)	138.1 (135.1, 141.4)	0.050
Potassium (mmol/L)	3.9 (3.6, 4.2)	3.9 (3.6, 4.2)	3.9 (3.6, 4.3)	0.593
WBC (× 10^9^/L)	6.1 (5.0, 7.8)	6.1 (5.0, 7.6)	6.3 (5.0, 8.3)	0.147
Total cholesterol (mmol/L)	3.60 (2.98, 4.40)	3.70 (3.00, 4.47)	3.54 (2.90, 4.32)	0.027
Triglyceride (mmol/L)	1.00 (0.77, 1.42)	1.02 (0.79, 1.47)	1.00 (0.75, 1.37)	0.054
HDL-C (mmol/L)	1.00 (0.79, 1.19)	1.00 (0.83, 1.22)	0.95 (0.74, 1.15)	< 0.001
LDL-C (mmol/L)	2.00 (1.48, 2.67)	2.02 (1.52, 2.76)	2.00 (1,43, 2.59)	0.117
AST (U/L)	23.2 (17.3, 32.6)	22.2 (16.8, 32.8)	24.0 (18.4, 35.7)	0.006
ALT (U/L)	19.5 (12.9, 32.6)	19.8 (12.9, 32.4)	19.3 (13.0, 33.4)	0.897
IVSTD (cm)	0.9 (0.8, 1.0)	0.9 (0.8, 1.0)	0.9 (0.8, 1.0)	0.440
LVPWTD (cm)	0.9 (0.8, 1.0)	0.9 (0.8, 1.0)	0.9 (0.8, 1.0)	0.480
LVDD (cm)	5.8 (5.2, 6.6)	5.9 (5.3, 6.6)	5.8 (5.1, 6.5)	0.776
LAD (cm)	4.8 (4.4, 5.3)	4.8 (4.4, 5.3)	4.8 (4.4, 5.3)	0.736
LVEF, n (%)				0.140
≤ 40	535 (51.9)	291 (53.1)	244 (50.5)	–
41–49	167 (16.2)	96 (17.5)	71 (14.7)	–
> 50	329 (31.9)	161 (29.4)	168 (34.8)	–
Medications at admission, n (%)				
Antiplatelet agent	455 (44.1)	116 (37.4)	205 (42.4)	0.305
ACEI/ARB/ARNI	406 (39.4)	237 (43.2)	169 (35.0)	0.007
Beta-blocker	807 (78.3)	433 (79.0)	374 (77.4)	0.539
Statins	561 (54.4)	304 (55.5)	257 (53.2)	0.466
Diuretics	914 (88.7)	270 (87.1)	428 (88.6)	0.970
Digoxin	122 (11.8)	50 (9.1)	72 (14.9)	0.004
Aldosterone antagonist	419 (40.6)	214 (39.1)	205 (42.4)	0.268
Insulin	134 (13.0)	77 (14.1)	57 (11.8)	0.284
Metformin	146 (14.2)	86 (15.7)	60 (12.4)	0.133
Sulfonylureas	9 (0.9)	7 (1.3)	2 (0.4)	0.137
SGLT2i	50 (4.8)	30 (5.5)	20 (4.1)	0.320

*MACCE* Major adverse cardiac and cerebrovascular events; BMI, Body mass index; *SBP* Systolic blood pressure; *DBP* Diastolic blood pressure; *FPG* Fasting plasma glucose; *FAR* Fibrinogen-to-albumin ratio; *BNP* B-type natriuretic peptide; *CRP* C-reative protein; *ALB* Albumin; *UA* Uric acid; *Cr* Creatinine; *Na* Sodium; *K* Potassium; *WBC* White blood cell; *HDL-C* High-density lipoprotein cholesterol; *LDL-C* Low-density lipoprotein-C; *AST* Alanine aminotransferase; *ALT* Aspartate transaminase; *IVSTD* Interventricular septal thickness at diastole; *LVPWTD* Left ventricular posterior wall end-diastolic thickness; *LVDD* Left ventricular end-diastolic diameter; *LAD* Left atrial diameter; *LVEF* Left ventricular ejection fraction; *ACEI* Angiotensin-converting enzyme inhibitor; *ARB* Angiotensin receptor inhibitor; *ARNI* Angiotensin receptor neprilysin inhibitor; *SGLT2i* Sodium-glucose cotransporter 2 inhibitor

### Comparison of clinical data among the two groups

**Fig. 2:**
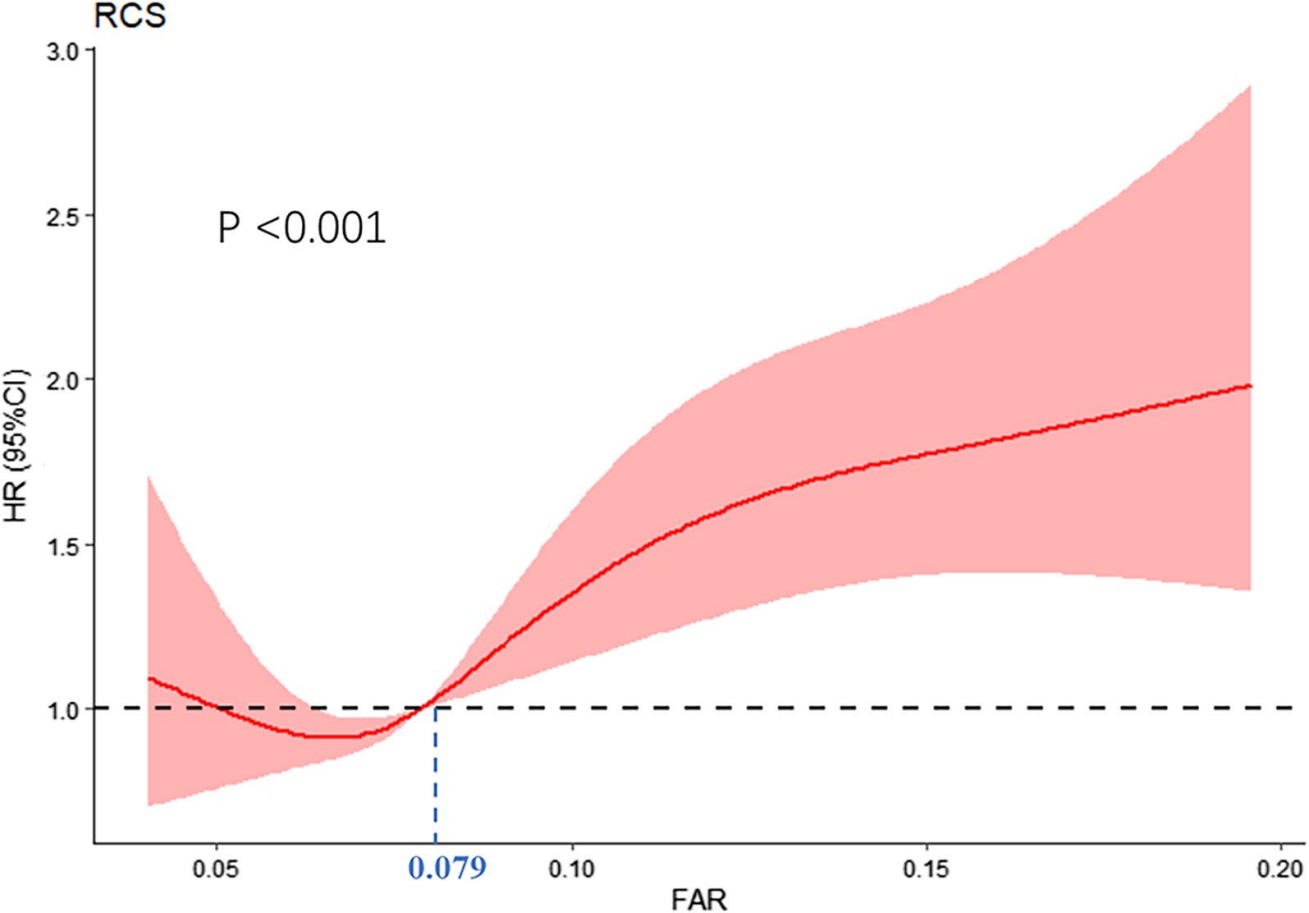
Hazard ratios for the MACCEs based on restricted cubic spine function for FAR. The red line represents the reference of the hazard ratios, and the red area represents 95% confidence intervals

Based on the restricted cubic spline analysis (Fig. 2), the optimal FAR cut-off value was 0.079 (p-value for non-linear was < 0.001). The patients were categorized into 2 groups based on the FAR value and the baseline characteristics of the two groups were analyzed (Table 2). In the FAR-H group, the fibrinogen, FPG, HbA1C, BNP, CRP, and WBC levels, the proportion of IMD, and occurrence of atrial fibrillation, hypertension, and the use of SGLT2i were significantly higher, compared to the FAR-L group. But the BMI, ALB, hemoglobin, hematocrit, sodium, HDL-C, AST, LVDD, LAD, the proportion of NIMD were relatively lower in the FAR-H group.

**Table 2:** Clinical characteristics according to the level of FAR

Characteristics	FAR-L(FAR < 0.079) (n=436)	FAR-H(FAR ≥ 0.079) (n=595)	p Value
Female, n (%)	168 (38.5)	225 (37.8)	0.815
Age (years)	69 (59, 78)	72 (64, 80)	0.097
BMI (kg/m^2^)	24.5 (21.6, 27.2)	24.0 (21.5, 27.0)	< 0.001
Heart rate (bpm)	80 (67, 98)	81 (70, 96)	0.126
SBP (mmHg)	127 (114, 142)	130 (115, 146)	0.092
DBP (mmHg)	75 (66,88)	75 (65,86)	0.141
Medical history, n (%)			
Ischemic cardiomyopathy	137 (38.5)	252 (42.5)	< 0.001
None-ischemic cardiomyopathy	117 (26.8)	92 (15.5)	< 0.001
Valvular heart disease	90 (20.6)	113 (19.0)	0.510
Atrial fibrillation	210 (48.2)	236 (39.7)	0.006
Hypertension	238 (54.6)	387 (65.0)	0.001
Hyperlipidemia	35 (8.0)	53 (8.9)	0.617
Glucose metabolism states			0.001
DM	184 (42.4)	321 (53.9)	
Pre-DM	146 (33.5)	163 (27.4)	
NGR	106 (24.3)	111 (18.7)	
Fibrinogen (g/L)	2.4 (2.1, 2.7)	3.5 (3.1, 4.1)	< 0.001
ALB (g/L)	39.6 (37.2, 41.7)	37.0 (34.2, 39.3)	< 0.001
FPG (mg/dL)	5.00 (4.58, 5.97)	5.2 (4.58, 6.58)	0.010
HbA1c (%)	6.0 (5.6, 6.7)	6.2 (5.7, 7.1)	0.008
Hemoglobin (g/L)	133 (119, 145)	125 (104, 138)	< 0.001
BNP (pg/mL)	422.0 (192.0, 990.8)	569.0 (242.0, 1220.0)	0.001
CRP (mg/L)	3.6 (2.5, 5.2)	7.0 (3.6, 24.7)	< 0.001
Hematocrit (%)	39.5 (35.8, 43.0)	37.7 (31.8, 41.3)	< 0.001
UA (μmol/L)	423.0 (331.5, 535.8)	437.0 (348.0, 548.0)	0.345
Creatinine (μmol/L)	79.0 (64.5, 100.1)	87.9 (69.0, 123.0)	< 0.001
Sodium (mmol/L)	138.8 (136.0, 141.6)	138.3 (135.0, 141.4)	0.030
Potassium (mmol/L)	3.9 (3.6, 4.2)	3.9 (3.6, 4.2)	0.725
WBC (× 10^9^/L)	5.9 (4.8,7.0)	6.5 (5.2, 8.3)	< 0.001
Total cholesterol (mmol/L)	3.63 (3.00, 4.38)	3.59 (2.94, 4.42)	0.751
Triglyceride (mmol/L)	1.00 (0.75, 1.52)	1.01 (0.78, 1.37)	0.455
HDL-C (mmol/L)	1.00 (0.82, 1.23)	0.98 (0.77, 1.14)	0.002
LDL-C (mmol/L)	2.00 (1.54, 2.62)	2.01 (1.43, 2.74)	0.760
AST (U/L)	24.2 (18.3, 34.0)	22.8 (16.7, 33.0)	< 0.001
ALT (U/L)	20.9 (14.7, 35.9)	17.9 (11.8, 31.0)	0.051
IVSTD (cm)	0.9 (0.8, 1.0)	0.9 (0.8, 1.1)	0.055
LVPWTD (cm)	0.9 (0.8, 1.0)	0.9 (0.8, 1.0)	0.092
LVDD (cm)	6.0 (5.2, 6.8)	5.8 (5.1, 6.5)	0.004
LAD (cm)	5.0 (4.4, 5.4)	4.8 (4.4, 5.2)	0.001
LVEF, n (%)			0.064
≤ 40	244 (56.0)	291 (48.9)	–
41–49	61(14.0)	106(17.8)	–
> 50	131(30.0)	198(33.3)	–
Medications at admission, n (%)			
Antiplatelet agent	182(40.1)	273(45.9)	0.186
ACEI/ARB/ARNI	175(40.1)	231(38.8)	0.670
Beta-blocker	336(77.1)	471(79.2)	0.420
Statins	241(55.3)	320(53.8)	0.634
Diuretics	388(89.0)	526(88.4)	0.769
Digoxin	50(11.5)	72(12.1)	0.756
Aldosterone antagonist	175(40.1)	244(41)	0.779
Insulin	62(14.2)	72(12.1)	0.371
Metformin	61(14.0)	85(14.3)	0.893
Sulfonylureas	3(0.7)	6(1.0)	0.585
SGLT2i	5(1.6)	39(6.6)	0.003

*BMI* Body mass index; *SBP* Systolic blood pressure; *DBP* Diastolic blood pressure; *FPG* Fasting plasma glucose; *FAR* Fibrinogen-to-albumin ratio; *BNP* B-type natriuretic peptide; *CRP* C-reative protein; *ALB* Albumin; *UA* Uric acid; *Cr* Creatinine; *Na* Sodium; *K* Potassium; *WBC* White blood cell; *HDL-C* high-density lipoprotein cholesterol; *LDL-C* Low-density lipoprotein-C; *AST* Alanine aminotransferase; *ALT* Aspartate transaminase; *IVSTD* Interventricular septal thickness at diastole; *LVPWTD* Left ventricular posterior wall end-diastolic thickness; *LVDD* Left ventricular end-diastolic diameter; *LAD* Left atrial diameter; *LVEF* Left ventricular ejection fraction; *ACEI* Angiotensin-converting enzyme inhibitor; *ARB* Angiotensin receptor inhibitor; *ARNI* Angiotensin receptor neprilysin inhibitor; *SGLT2i* Sodium-glucose cotransporter 2 inhibitor

### Prediction of the incidence of MACCEs by FAR

**Fig. 3:**
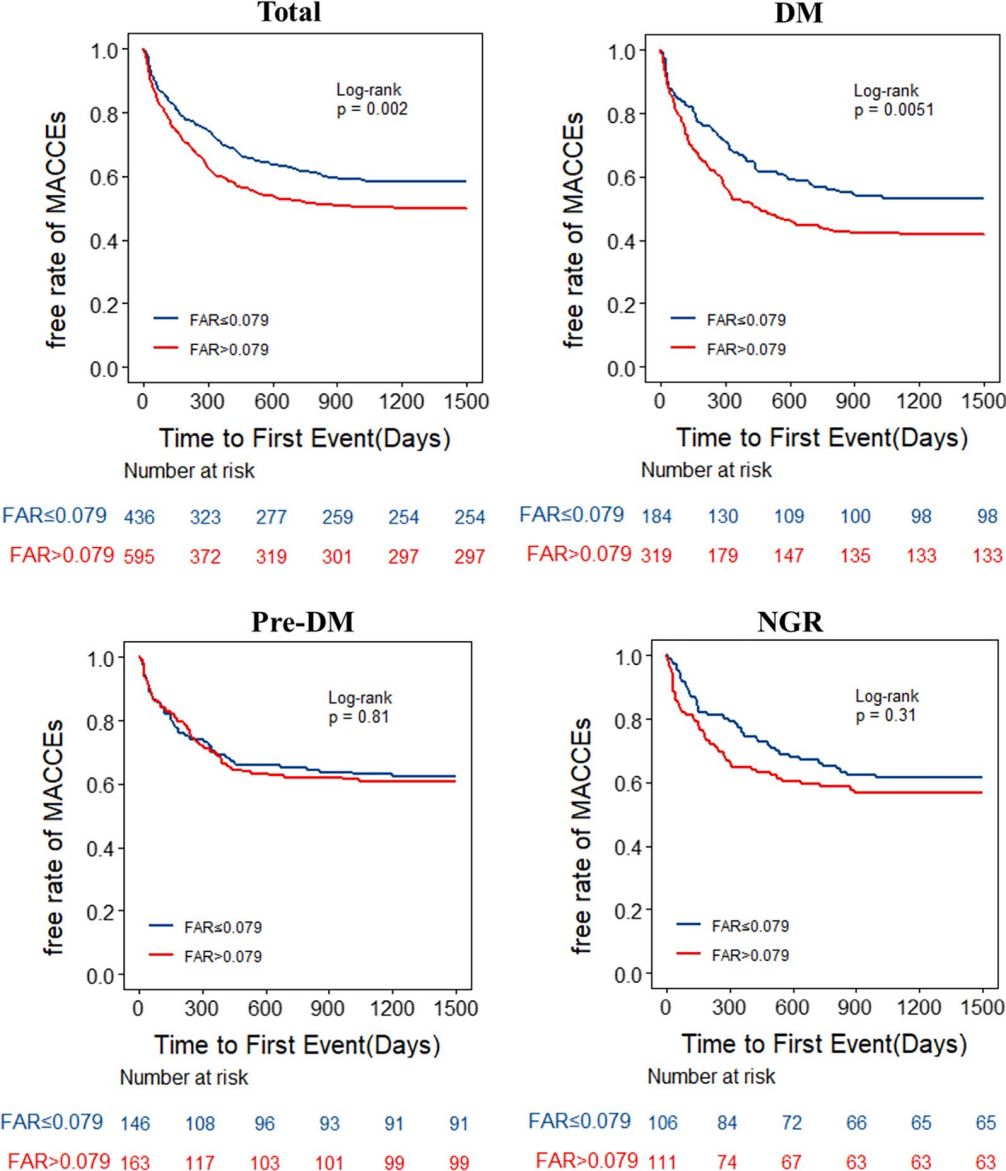
The Kaplan-Meier analysis for MACCEs according to different FAR levels for total patients (**A**), patients with DM (**B**), patients with Pre-DM (**C**) and patients with NGR (**D**)

The FAR was significantly associated with the incidence of MACCEs in all participants, as shown in Fig. 3A. Table 3 shows three multivariate Cox proportional hazard models to determine the correlation between the FAR groups and MACCEs. Consequently, high FAR was associated with higher incidence of MACCEs (Model 1, HR = 1.38, 95% CI 1.14–1.66, p = 0.001; Model 2, HR = 1.29, 95% CI 1.07–1.55, p = 0.008; Model 3, HR = 1.29, 95% CI 1.07–1.56, p = 0.007).

**Table 3:** The HR (95% CI) of MACCEs according to FAR and different glucose metabolism states from the three models

Glucose regulation state	FAR	Events, n (%)	Model 1		Model 2		Model 3	
			HR (95% CI)	p-Value	HR (95% CI)	p-Value	HR (95% CI)	p-Value
Total	Low	182 (17.7)	Ref.		Ref.		Ref.	
	High	301 (29.2)	1.32 (1.09–1.59)	0.003	1.29 (1.07–1.55)	0.008	1.29 (1.07–1.56)	0.007
DM	Low	86 (8.3)	Ref.		Ref.		Ref.	
	High	189 (18.3)	1.52 (1.17–1.96)	0.002	1.46 (1.13–1.89)	0.004	1.48 (1.14–1.92)	0.003
Pre-DM	Low	55 (5.3)	Ref.		Ref.		Ref.	
	High	64 (6.2)	1.01 (0.70–1.45)	0.946	0.96 (0.66–1.39)	0.814	0.95 (0.65–1.39)	0.799
NGR	Low	41 (4.0)	Ref.		Ref.		Ref.	
	High	48 (4.7)	1.18 (0.77–1.82)	0.432	1.13 (0.71–1.79)	0.591	1.10 (0.69–1.73)	0.693

Model 1: Adjusted for gender, age, BMI, SBP, DBP, and heart rate. Model 2: Adjusted for Model 1 + BNP, creatinine, WBC, sodium, UA, ALT, and LVEF. Model 3: Adjusted for Model 2 + history of hypertension, valvular heart disease, atrial fibrillation, and use of aldosterone antagonist, β-Blockers, ACEI/ARB/ARNI, and SGLT2i

*CI* Confidence interval; *FAR* Fibrinogen-to-albumin ratio; *HR* Hazard ratio

### Associations between FAR and MACCEs at different glucose metabolism states

**Fig. 4:**
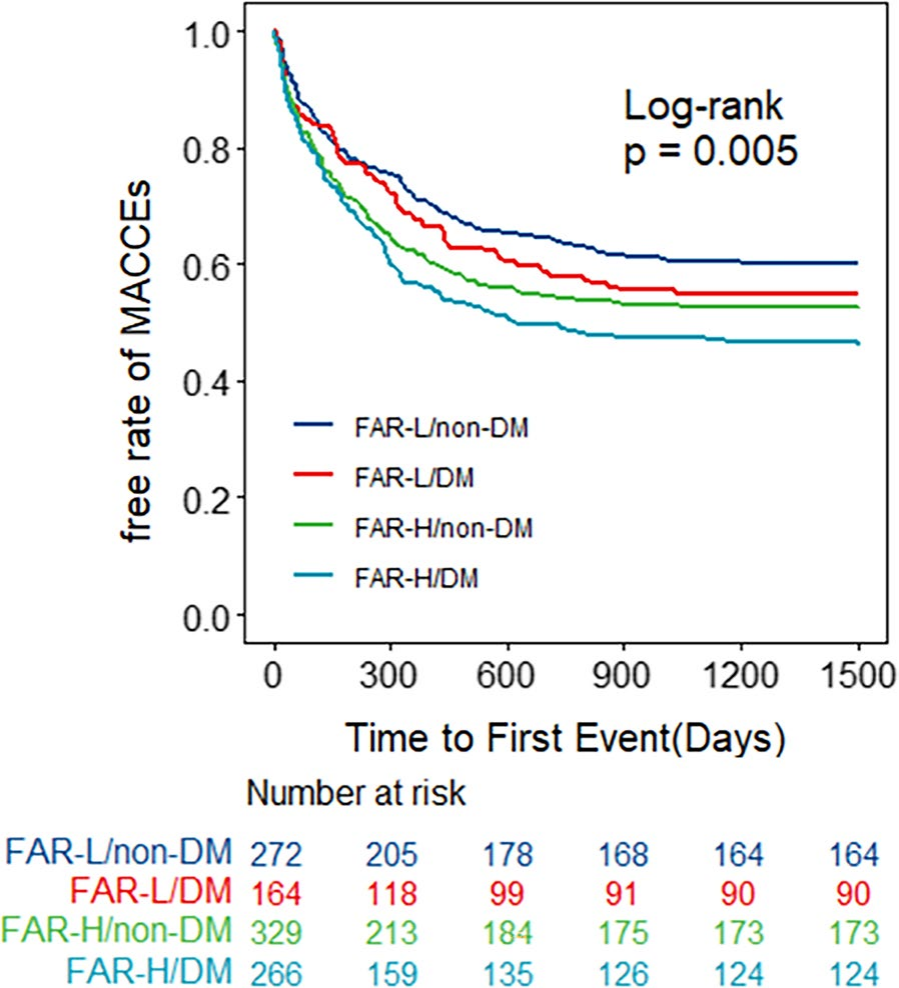
The Kaplan-Meier curve analysis for MACCEs according to status of FAR and glycemic metabolism

Specific to different glucose metabolism states, the Kaplan–Meier curves (Fig. 3B–D) showed a significant difference in the incidence of MACCEs for DM between the FAR groups (P = 0.005), while no significant difference was found for pre-DM and NGR (all p-values were > 0.05). The high FAR was associated with MACCEs for DM in three multivariate Cox models (Model 1, HR = 1.52, 95% CI 1.17–1.96, p = 0.002; Model 2, HR = 1.46, 95% CI 1.13–1.89, p = 0.004; Model 3, HR = 1.48, 95% CI 1.14–1.92, p = 0.003; Table 3). But no significant differences were found between the FAR groups for pre-DM and NGR in three Cox models (all p-values were > 0.05). The FAR-H-DM combination group had the highest risk of developing MACCEs compared with the other groups (p = 0.005) (Fig. 4).

### The correlation between FAR and other cardiometabolic factors

The Spearman’s rank correlation analysis outcomes are reported in Table 4. The FAR was positively correlated with age, CRP, WBC, BNP, FPG, and HbA1C, but negatively correlated with BMI and HDL-C (all p-values were < 0.05).

**Table 4:** The correlation coefficients between FAR and other cardiometabolic factors

Variables	Coefficient (r)	p-Value	
Age	0.104	0.001	
BMI	− 0.062	0.045	
CRP	0.481	< 0.001	
WBC	0.226	< 0.001	
BNP	0.128	< 0.001	
HDL-C	− 0.114	< 0.001	
FPG	0.124	< 0.001	
HbA1C	0.105	0.001	

*FAR* Fibrinogen-to-albumin ratio; *BMI* Body mass index; *CRP* C Reactive protein; *WBC* White blood cell; *BNP* B-type natriuretic peptide; *HDL-C* High-density lipoprotein-C; *FPG* Fasting plasma glucose

### Subgroup analysis

**Fig. 5:**
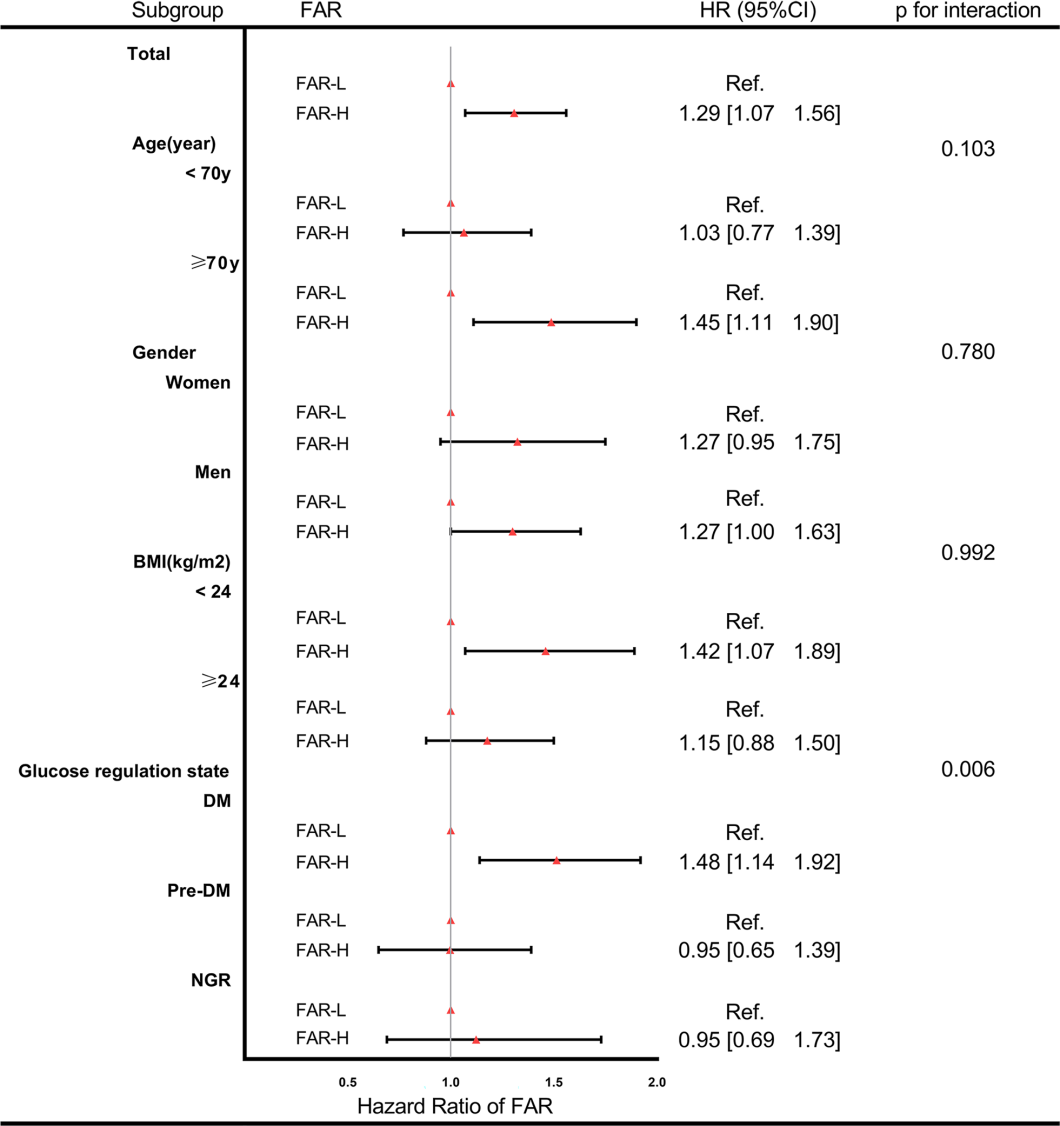
The forest plot of MACCEs specific to different subgroups. Adjusted model included FAR, age, sex, body mass index, systolic blood pressure, diastolic blood pressure, heart rate, BNP, sodium,creatinine, UA, WBC, ALT, LVEF, history of hypertension, valvular heart disease, atrial fibrillation, aldosterone antagonist, β-blockers, ACEI/ARB/ARNI, and SGLT2i use

On post-hoc subgroup analysis, the FAR-H was associated with a high incidence of MACCEs, which was consistent across subgroups, age, sex, and BMI (Fig. 5). There were no interactions between the FAR and the above variables in the subgroup analyses (all p-values for interaction were > 0.05). But interaction between FAR and glucose metabolism state was seen (p-value for the interaction = 0.006). In the DM group, FAR-H was associated with the high risk of MACCEs, but not in the other groups.

## Discussion

In this retrospective study, the relationship between FAR, a simple proportion, and the risk of MACCEs in patients with ADHF was explored in a clinical setting. Upon analysis, the FAR was positively correlated with the indicators of glucose metabolism, such as HbA1C and FPG. Moreover, high FAR was significantly associated with the increased risk of MACCEs in patients with ADHF and DM. However, we did not find a significant correlation in patients with ADHF at pre-DM state.

### The association between FAR and CVD risk

FAR, as a proportion of fibrinogen and albumin, has been elucidated in several diseases. Numerous studies demonstrated that preoperative FAR was associated with the adverse prognosis after surgery in many cancers, such as hepatocellular carcinoma, cholangiocarcinoma, and glioblastoma [28–31]. Additionally, FAR has been frequently studied in cardiovascular diseases (CVD), particularly CAD. FAR was a useful tool in predicting the severity of CAD in patients with the acute coronary syndrome (ACS) [32–35]. FAR was associated with the long-term prognosis in patients with ST-elevation myocardial infarction (STEMI), non-STEMI, and CAD who underwent percutaneous coronary intervention (PCI) [35–38]. Park et al. found FAR as a prognostic marker of high mortality in patients with CAD who underwent off-pump coronary artery bypass grafting [39].

### Plausible association between FAR and ADHF with the DM state

Fibrinogen, an inflammatory marker [40], was increased in chronic inflammatory conditions such as diabetes, atherosclerosis, and HF [10, 41, 42]. The fibrinogen was related to the high risk of HF incidence [43, 44]. Meng et al. reported that high levels of fibrinogen displayed a higher risk of 90-day mortality in patients with ADHF [45]. Albumin was considered an important biomarker of malnutrition and inflammation in patients with HF [46]. Hypoalbuminemia was a predictor of short-term prognosis in patients with acute heart failure [46].

Wang et al. showed that a higher level of FAR with the DM state was associated with the worse 5-year outcome in patients with CAD undergoing PCI [20]. The role of chronic and low-grade inflammation in HF with DM was demonstrated, and DM as comorbidity contributed to the worse prognosis in patients with HF [4, 7, 23]. However, FAR has not yet been correlated with HF at the DM state, especially ADHF, which is a highly heterogeneous syndrome [47, 48]. In this study, the significant predictive value of FAR for developing MACCEs was demonstrated in patients with ADHF and DM, for the first time.

### Potential mechanisms between FAR and ADHF

The underlying mechanism between FAR and ADHF needs to be elucidated. Acute phase reactants (APR) are inflammatory markers, which are produced in the liver in response to interleukin-6 (IL-6) [49]. APR can be upregulated or downregulated, with increasing or decreasing concentration during inflammation [49]. Positive APR includes procalcitonin, C-reactive protein, and fibrinogen, and negative APR includes albumin, prealbumin, and transferrin [49]. When acute exacerbation of chronic HF occurs, protein metabolism in the liver may synthesize pro-inflammatory proteins such as fibrinogen, emphasizing the role of a positive APR. Meanwhile, the hypoalbuminemia in the physiopathology of CVD may be from anti-inflammatory, antioxidant, anticoagulant, and anti-aggregation responses. Hypoalbuminemia was associated with long-term protein dystrophy in chronic HF [50], and albumin may have been further affected by the inflammatory status in patients with ADHF. Thus, when patients are with ADHF at the DM state, the higher the FAR level and the higher the degree of inflammation that may occur.

### FAR as a potential therapeutic target

Previous research demonstrated that fibrinogen up-regulated the expression of proinflammatory cytokines, such as interleukin-1 (IL-1) and tumor necrosis factor-α (TNF-α) [51]. In the CANTOS study, IL-1β targeting Canakinumab significantly reduced the incidence of MACE in HF patients [7]. Inhibition of fibrinolysis by drugs, such as tranexamic acid, reduced inflammatory response in patients who underwent cardiopulmonary bypass [52]. However, no evidence currently exists that reducing fibrinogen or correcting hypoalbuminemia improves the prognosis of CVD. Therefore, the potential mechanisms and targeted therapeutic strategies of FAR in ADHF with DM state should be further investigated.

## Limitations

This study also had several limitations. First, due to the retrospective design, the FAR was not dynamically measured in patients over the follow-up period. Second, as this was a single-center study with limited sample size, data bias could not be avoided despite correcting multiple confounding factors. Third, prospective cohort studies are required to validate these findings.

## Conclusion

This study demonstrated that FAR was independently associated with the adverse prognosis in patients with ADHF at DM state, but this association was not observed in patients at pre-DM and NGR states.

## Data Availability

The information and data of the study population were extracted from the hospital information system. The datasets are not publicly available because the privacy of the participants should be protected. Data are however available from the corresponding author on a reasonable request.

## Abbreviations

HF: Heart failure
CAD: Coronary artery disease
FAR: Fibrinogen-to-albumin ratio
DM: Diabetes mellitus
MACE: Major adverse cardiovascular events
MACCEs: Major adverse cardiac and cerebral events
ADHF: Acute decompensated heart failure
BMI: Body mass index
FPG: Fasting plasma glucose
HbA1c: Glycated hemoglobin
Pre-DM: Prediabetes mellitus
NGR: Normal glucose regulation
SBP: Systolic blood pressure
DBP: Diastolic blood pressure
TC: Total cholesterol
LDL-C: Low-density lipoprotein-C
HDL‑C: High‑density lipoprotein cholesterol
ALB: Albumin
CRP: C-reactive protein
BNP: B-type natriuretic peptide
UA: Uric acid
WBC: White blood cell
ALT: Aspartate transaminase
AST: Alanine aminotransferase
SD: Standard deviation
IQR: Interquartile range
ANOVA: Analysis of variance
LVEF: Left ventricular ejection fraction
HRs: Hazard ratios
CIs: 95% Confidence intervals NIMD: none-ischemic cardiomyopathy
ACEI: Angiotensin-converting enzyme inhibitor
ARB: Angiotensin receptor inhibitor
ARNI: Angiotensin receptor enkephalinase inhibitor
SGLT2i: Sodium-glucose contrasporter-2 inhibitor
CVD: Cardiovascular diseases
PCI: Percutaneous coronary intervention
APR: Acute phase reactants
IL-1: Interleukin-1
TNF-α: Tumor necrosis factor-α
